# Leukocyte filtration and leukocyte modulation therapy during extracorporeal cardiopulmonary resuscitation in a porcine model of prolonged cardiac arrest

**DOI:** 10.1038/s41598-024-63522-w

**Published:** 2024-06-07

**Authors:** Jensyn J. VanZalen, Takahiro Nakashima, Annie Phillips, Joseph E. Hill, Angela J. Westover, Liandi Lou, Jinhui Liao, Joshua Mergos, Garrett Fogo, Thomas H. Sanderson, William C. Stacey, Mohamad Hakam Tiba, David H. Humes, Robert H. Bartlett, Alvaro Rojas-Peña, Robert W. Neumar

**Affiliations:** 1 Department of Surgery and ECLS Laboratory, University of Michigan Medical School, Ann Arbor, MI 48109 USA; 2 The Max Harry Weil Institute for Critical Care Research and Innovation, University of Michigan, Ann Arbor, MI 48109 USA; 3 Department of Internal Medicine, University of Michigan Medical School, Ann Arbor, MI 48109 USA; 4 Department of Emergency Medicine, University of Michigan Medical School, 1500 E Medical Center Drive, Ann Arbor, MI 48109-5303 USA; 5 Movement Science, University of Michigan School of Kinesiology, Ann Arbor, MI 48109 USA; 6 Department of Neurology, University of Michigan Medical School, Ann Arbor, MI 48109 USA; 7 Neuroscience Graduate Program, University of Michigan Medical School, Ann Arbor, MI 48109 USA; 8 Department of Molecular and Integrative Physiology, University of Michigan Medical School, Ann Arbor, MI 48109 USA; 9 Department of Surgery Section of Transplantation, University of Michigan Medical School, Ann Arbor, MI 48109 USA

**Keywords:** Extracorporeal cardiopulmonary resuscitation, Cardiac arrest, Inflammation, Leukocyte modulation, Neutrophil extracellular traps, Animal models, NETosis, Cardiac device therapy, Experimental models of disease, Translational research, Brain injuries, Hypoxic-ischaemic encephalopathy, Neurodegeneration, Preclinical research, Inflammation

## Abstract

Extracorporeal cardiopulmonary resuscitation (ECPR) is emerging as a feasible and effective rescue strategy for prolonged cardiac arrest (CA). However, prolonged total body ischemia and reperfusion can cause microvascular occlusion that prevents organ reperfusion and recovery of function. One hypothesized mechanism of microvascular “no-reflow” is leukocyte adhesion and formation of neutrophil extracellular traps. In this study we tested the hypothesis that a leukocyte filter (LF) or leukocyte modulation device (L-MOD) could reduce NETosis and improve recovery of heart and brain function in a swine model of prolonged cardiac arrest treated with ECPR. Thirty-six swine (45.5 ± 2.5 kg, evenly distributed sex) underwent 8 min of untreated ventricular fibrillation CA followed by 30 min of mechanical CPR with subsequent 8 h of ECPR. Two females were later excluded from analysis due to CPR complications. Swine were randomized to standard care (Control group), LF, or L-MOD at the onset of CPR. NET formation was quantified by serum dsDNA and citrullinated histone as well as immunofluorescence staining of the heart and brain for citrullinated histone in the microvasculature. Primary outcomes included recovery of cardiac function based on cardiac resuscitability score (CRS) and recovery of neurologic function based on the somatosensory evoked potential (SSEP) N20 cortical response. In this model of prolonged CA treated with ECPR we observed significant increases in serum biomarkers of NETosis and immunohistochemical evidence of microvascular NET formation in the heart and brain that were not reduced by LF or L-MOD therapy. Correspondingly, there were no significant differences in CRS and SSEP recovery between Control, LF, and L-MOD groups 8 h after ECPR onset (CRS = 3.1 ± 2.7, 3.7 ± 2.6, and 2.6 ± 2.6 respectively; *p* = 0.606; and SSEP = 27.9 ± 13.0%, 36.7 ± 10.5%, and 31.2 ± 9.8% respectively, *p* = 0.194). In this model of prolonged CA treated with ECPR, the use of LF or L-MOD therapy during ECPR did not reduce microvascular NETosis or improve recovery of myocardial or brain function. The causal relationship between microvascular NETosis, no-reflow, and recovery of organ function after prolonged cardiac arrest treated with ECPR requires further investigation.

## Introduction

Extracorporeal cardiopulmonary resuscitation (ECPR) using percutaneous venoarterial extracorporeal membrane oxygenation (ECMO) is emerging as a feasible and effective resuscitation strategy for patients who fail to achieve return of spontaneous circulation (ROSC) with standard resuscitation efforts. For patients with witnessed refractory out-of-hospital cardiac arrest (OHCA), reported survival rates with ECPR in randomized clinical trials range from 0 to 40%^1–4^.

Intravascular complications of total-body ischemia and reperfusion, including microvascular coagulation, leukocyte adhesion, and neutrophil extracellular trap (NET) formation may cause no-reflow and prevent recovery of heart and brain function when ECPR is used to treat prolonged cardiac arrest (CA)^5–8^. In particular, leukocyte-mediated response during and after CA has been implicated in tissue injury and multi-organ dysfunction in a whole body, similar to a systemic inflammatory response^9–11^. In addition, extracorporeal support is known to activate leukocytes by contact with the foreign and non-endothelialized surfaces of the extracorporeal circuits^12,13^.

In this study, we investigated two strategies to mitigate leukocyte activation and NETosis, a leukocyte filter (LF) and a leukocyte modulation device (L-MOD). The LF mitigates leukocyte-mediated injury by trapping leukocytes^14^. The mechanism by which the LF removes circulating leukocytes from the blood is adhesion to the filter membrane. In patients who underwent cardiac surgery with cardiopulmonary bypass, several studies have reported the beneficial effect of a LF on reducing myocardial^13,14^ or cerebral damage^15,16^ whereas others have shown no benefits^17–19^. The second strategy investigated the efficacy of the L-MOD in tandem with the extracorporeal circuit. L-MOD therapy incorporates a selective cytopheretic device (SCD) with regional citrate anticoagulation (RCA)^20^. L-MOD sequesters activated leukocytes using low shear flow and by mimicking surface characteristics which encourage binding of activated cells, thus inhibiting the inflammatory activity of these cells^21^. The L-MOD is expected to alter the natural inflammatory process by preferentially binding and inactivating the activated leukocytes, unlike the standard LF, which, when incorporated into an extracorporeal circuit, traps leukocytes. We previously reported that the L-MOD ameliorates the multi-organ dysfunction effects of systemic inflammatory response syndrome and has an impact on the mortality rate of multi-organ failure in intensive care unit patients^22–24^.

The aim of the present study was to compare the effect of a standard LF system and a L-MOD device during ECPR on NETosis and recovery of cardiac and neurologic function after prolonged CA using a swine model. We hypothesized that LF or L-MOD would reduce neutrophil activation and microvascular NET formation, improving recovery of heart and brain function.

## Methods

Animal procedures were performed at the University of Michigan Extracorporeal Life Support Laboratory in accordance with the ARRIVE guidelines. Animal husbandry and veterinary care were provided by the Unit for Laboratory Animal Medicine (ULAM) under the guidance of animal technologists certified by the American Association for Laboratory Animal Science. Animals in this experiment were treated in compliance with “The Guide for Care and Use of Laboratory Animals'' National Research Council of the National Academies 8th edition (revised 2011). Protocol #00011170 was approved on 1/17/2023 by the University of Michigan Institutional Animal Care and Use Committee. Animal preparation and methodology were adapted from the first phase of our study as described in VanZalen et al.^25^.

### Animal preparations

Thirty-six swine (Yorkshire, 40–50 kg, 2–3 months old, evenly distributed sex) were studied. Animals with a white blood cell count greater than 20,000 K/uL on experiment day were excluded to avoid active infection and not included as a part of the total prepared n = 36. Swine were block randomized into one of the three following groups: standard care ECPR (Control group); ECPR with LF (LF group), or ECPR with L-MOD (L-MOD group). In this study, experimental operators were not blinded.

Animals were initially sedated with 5 mg/kg tiletamine and zolazepam and 3 mg/kg xylazine, and anesthesia was induced with inhaled isoflurane (1–3%) with standard volume setting: tidal volume 7–9 mL/kg, respiratory rate adjusted to maintain a PaCO_2_ of 40 ± 5 mmHg, FiO_2_ 0.21–0.5 (adjusted to maintain PaO_2_ between 100 and 200 mmHg), PEEP 5 cmH_2_O. Core temperature was maintained between 37 and 37.5 °C. After central lines were placed and secured, total IV anesthesia was implemented as described in our previous work^25^ 60 min prior to initiation of the study.

### Hemodynamic monitoring

Detailed preparation of hemodynamic monitoring was previously described^25^. Lead II electrocardiography (ECG) was continuously recorded. Monitoring lines were placed in the right subclavian artery for arterial pressure (AP) and vein for central venous pressure (CVP). An 8 Fr pulmonary artery catheter (PAC, Edwards LifeSciences LLC, Irvine, CA) was inserted for continuous monitoring of mixed venous oxygen saturation (SvO_2_) and cardiac output (CO) by thermodilution technique. A multichannel Data Acquisition System (MP150, Biopac, Goleta, CA) recorded all hemodynamic monitoring throughout the CA period. Prior to initiation of CA, surgical cut downs were performed to place 4 Fr percutaneous sheath catheters (Boston Scientific, M00115710B1) in the groin to expose the right femoral artery and left femoral vein in preparation for ECPR cannulation. Core temperature was maintained between 37 and 37.5 °C and monitored using a rectal temperature probe.

### Neurologic monitoring

Detailed preparation of neurologic monitoring was previously described in VanZalen et al.^25^. Cortical EEG activity and cerebral/somatic regional oxyhemoglobin saturation (rSO_2_) by near infra-red-light spectroscopy (INVOSTM5100 Cerebral/Somatic Oximeter. Covidien, Mansfield, MA) were continuously recorded. Somatosensory evoked potentials (SSEPs) were measured and recorded trans-cranially using corkscrew electrodes placed in a modified 10–20 montage from the cortex, subcortical structures, and the right brachial plexus in response to median nerve stimulation at the forelimb (Cascade Elite, Cadwell, Kennewick, WA).

### Prolonged CA and CPR protocol

**Figure 1:**
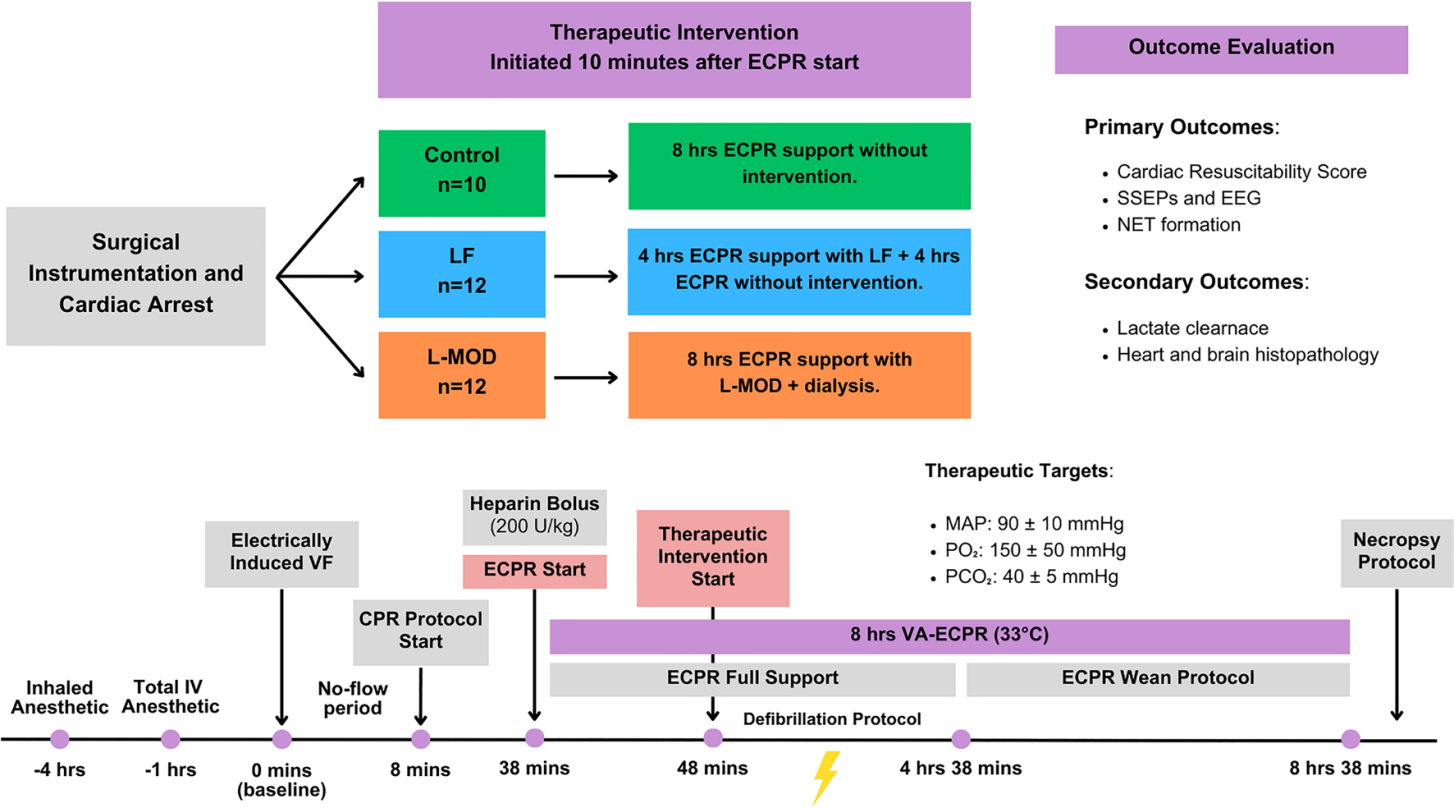
Study Design and Randomized Groups. After 8 min of untreated VF cardiac arrest, animals in all three groups underwent the same 30-min CPR protocol. At minutes 12, 16, and 20 all animals received epinephrine boluses (0.015 mg/kg). At minute 24, all animals received a vasopressin bolus (20 U). ECPR was initiated after 38 min and a heparin bolus of 200 U/kg was given. Ten minutes following ECPR initiation, the therapeutic intervention started (Control, LF, or L-MOD + dialysis). All three groups underwent the same defibrillation protocol (200 J × 3 shocks/hr) during the first four hours of ECPR support until ROSB was achieved. If ROSB was achieved, a weaning protocol was followed during the remaining four hours of ECPR. Hemodynamic targets were maintained throughout ECPR with vasopressor support of epinephrine (0.1–0.5 mcg/kg/min) and vasopressin (0–0.01 U/kg/min) during full ECMO support and norepinephrine (0.01–0.5 mcg/kg/min) and dobutamine (2–40 mcg/kg/min) during the ECMO weaning phase. At the end of the study, a necropsy was performed on all animals. Outcome evaluation focused on cardiac and cerebral recovery.

A transvenous right ventricular pacing wire was placed for induction of ventricular fibrillation (VF) using a right ventricular electrical stimulation (9v DC current)^26^ and confirmed by the ECG and loss of aortic pressure pulsations. Ventilation, anesthetics, fluids, and intraoperative warming devices were paused. Normothermic VF was left untreated for 8 min followed by a goal-directed, mechanical CPR (Lucas II, Stryker Medical, Kalamazoo, MI) at a depth of 5 cm and rate of 100/minute, and asynchronous mechanical ventilation with a tidal volume of 10 mL/kg, respiratory rate of 10, FiO_2_ 1.0, and PEEP of 5 cmH_2_O. Additionally, epinephrine (0.015 mg/kg) was administered at minutes 12, 16, and 20 following CA, with a vasopressin bolus (20 U) at minute 24. Cannulation of the femoral vessels at minutes 18 through the groin incision using a 20–22 Fr venous cannula (Avalon Elite Multi-Port Venous Femoral Catheter, MAQUET, Rastatt Baden-Württemberg, Germany) for blood drainage and a 15–17 Fr arterial cannula (NovaLung, Xenios, Heilbronn, Germany) for blood reinfusion. If end-tidal CO_2_ values reached less than 10 mmHg for greater than 10 min, ECPR was not initiated. The experimental timeline is shown in Fig. 1.

### Leukocyte modulating therapies

**Figure 2:**
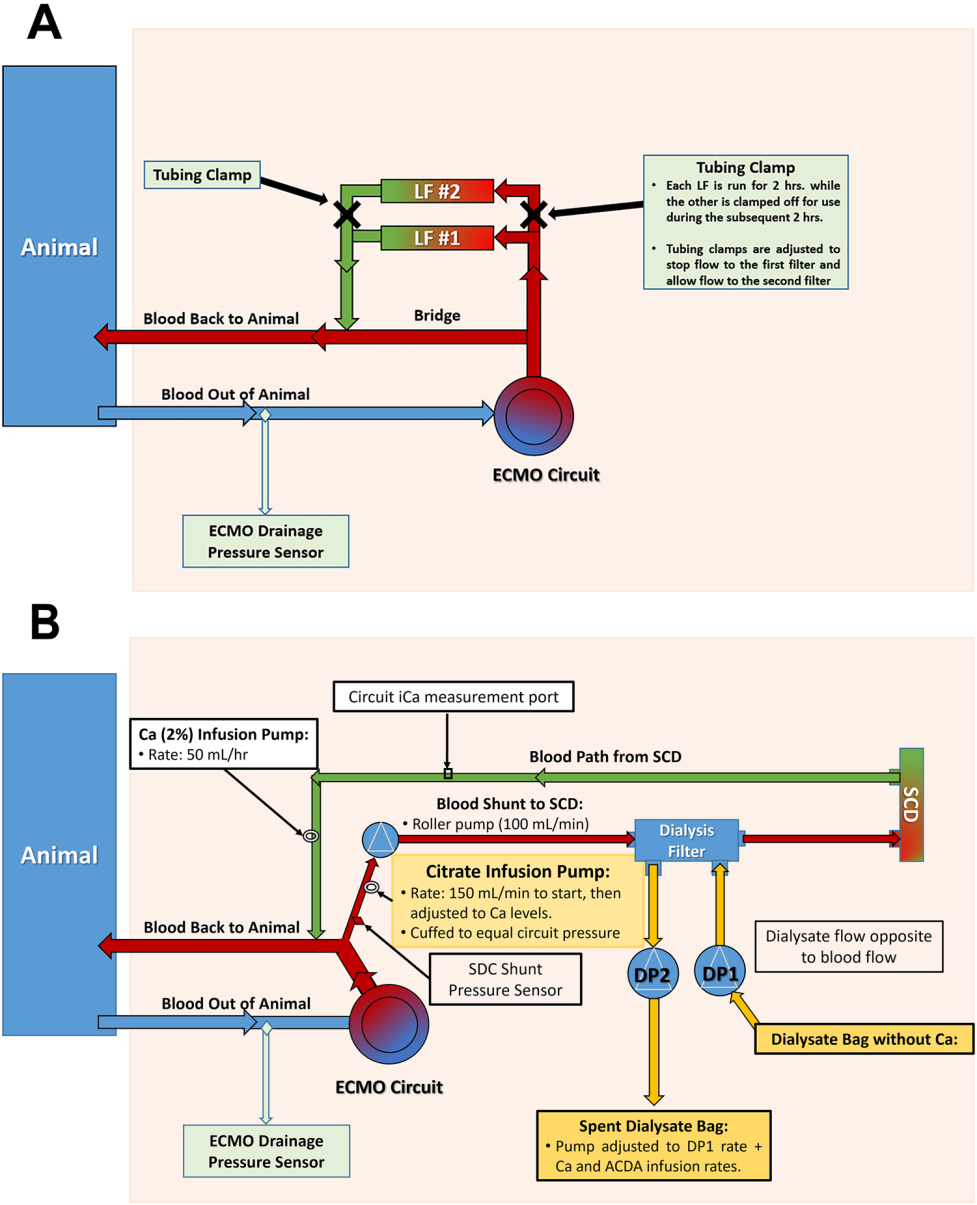
Schematic of the In-line Connection of the SCD System and LF to the ECMO circuit. (**A**) LF circuit configuration. LF is in-line with the ECMO circuit for the first four hours of ECPR support. A shunt from the post-oxygenator of the ECMO circuit connects the first and second PALL LeukoGuard® LS leukocyte reduction filters (East Hills, NY). Each filter is used for two hours. A tubing clamp is placed to prevent flow into the second filter until the first two hours are completed and the clamps are then adjusted to prevent flow to the first filter for the subsequent two hours. After four hours, clamps are placed to stop flow to both LFs and the circuit shunts from the oxygenator straight to the patient via the bridge depicted above. (**B**) L-MOD circuit configuration. First, a small shunt from the high-pressure part of the ECMO circuit post-oxygenator is used for SCD therapy using a roller pump that controls SCD blood flows (100 mL/min). A citrate infusion (150 mL/min) is given to chelate Ca pre-pump. Then, excess fluid is removed with a dialysis filter prior to SCD treatment. Once blood passes through the SCD cartridge iCa is measured and a Ca (2%) infusion is replaced prior to returning the blood to the animal to avoid systemic hypocalcemia.

The LF (PALL LeukoGuard® LS leukocyte reduction filter, East Hills, NY) was inserted in the arterial (re-infusion) line of the ECMO circuit to reduce the number of circulating neutrophils.Two filters were configured in parallel and placed in-line with the arterial outlet of the ECPR system (post-oxygenator). LF therapy was initiated 10 min after the onset of ECPR, and maintained during the first 4 h of support, switching from the first to the second filter after two hours to prevent filter saturation. 100% of ECPR blood flow was passed through the filters during the first 4 h of support and the shunt was clamped off. The filters were subsequently bypassed after the 4 h of support using the shunt to avoid oversaturation with leukocytes, and therefore high pressures in the arterial line (Fig. 2A).

The L-MOD was supplied by the David Humes Research Lab at the University of Michigan and the methodology for this study was adapted from Yessayan et al.^27^, in collaboration with the Humes Lab. The L-MOD is an extracorporeal cartridge containing polysulfone hollow fibers with a surface area of 2.5 m^2^ and is equivalent to the SCD 2.5^27^. Blood flow is directed to the side ports of the cartridge with closed end caps and enters the extra-capillary space of the device so that blood flows along the extraluminal side of the membranes with shear stress approaching capillary shear. This low shear stress environment allows activated leukocytes to adhere to the outer surface of the hollow fiber membranes while they traverse along the blood flow path.

The L-MOD treatment requires regional citrate anticoagulation (RCA) to maintain the ionized calcium (iCa) concentration in the blood circuit between 0.25 and 0.4 mM. This configuration allows continuous cell processing of circulating neutrophils and monocytes to a less proinflammatory phenotype, thereby tempering the hyperinflammatory state of the effector cells of the innate immunologic system which are central to promoting the cytokine storm and subsequent tissue damage. The L-MOD sustains the same blood flow settings of the continuous renal replacement therapy (CRRT) circuit. To attain its immunomodulatory effect, the L-MOD requires a rigorously controlled low ionized calcium (iCa) concentration between 0.25 and 0.4 mM in the extracorporeal blood circuit achieved with regional citrate anticoagulation (RCA) and dialysate/replacement fluid with no calcium. As required in RCA protocol, replacement calcium is infused into the blood circuit exiting the L-MOD so that the patient maintains a normal systemic calcium level.

The L-MOD is placed in-line with the arterial outlet of the ECPR system (post-oxygenator) and paired with dialysis filtration (Fig. 2B). The L-MOD was initiated 10 min after the start of ECPR and remained within the extracorporeal circuit for the remainder of the study (8 h).

### ECPR protocol

The ECPR system used was a roller peristaltic pump (COBE Perfusion System Precision Pump 043600-800; Terumo, Lakewood, CO). ECPR began 38 min after CA initiation. Initial ECPR flow was increased up to baseline CO and adjusted to achieve preset physiologic targets: MAP greater than 80 mmHg, PaO_2_ 150 ± 50 mmHg, and temperature-corrected PaCO_2_ 40 ± 5 mmHg. Epinephrine (0.1–0.5 mcg/kg/min) and vasopressin (0.002–0.01 U/kg/min) were used for pressor support during the first four hours of ECMO. After 15 min of ECPR support, external electrical defibrillation was attempted utilizing up to 3 shocks at 200 J with a biphasic defibrillator (Philips Medical System, Andover, MA). If electrical shock was unsuccessful and VF persisted, repeat defibrillation was attempted every 15 min throughout the first hour of ECPR support. If VF recurred after initial successful defibrillation, up to 3 defibrillation attempts (200 J) were performed for each episode of VF. In animals with ROSB, the ability to wean was assessed after 4 h of ECPR support with progressive decreased ECPR flow. Norepinephrine (0.01–0.5 mcg/kg/min) and dobutamine (2–40 mcg/kg/min) were used for hemodynamic support during ECPR weaning. Weaning was deemed successful if a stable MAP > 65 mmHg could be maintained with negligible ECPR flow (< 0.5 L/min). If swine were not able to maintain MAP greater than 65 mmHg despite interventions, the study was terminated. A neurological exam was performed every 30 min while on ECPR and if signs of life were observed, a propofol dose of 1–12 mg/kg/hr was administered intravenously for sedation.

### Data collection

Data of transthoracic echocardiography (TTE) and whole blood samples were collected at baseline, after ECPR initiation, 30 min after ECPR initiation, and then every hour until the end of the experiment. Left ventricular ejection fraction (LVEF) was calculated by the Teicholz method using TTE with a 2.18 Hz transducer (iU22, Philips, Amsterdam, Netherlands). Complete blood count and chemistry analysis were performed using Procyte DX and Catalyst DX (Idexx Laboratories, Westbrook, ME). Coagulation parameters were assessed using the BCS XP coagulation analyzer (Siemens, Malvern, PA). Blood gas analysis was performed with Radiometer of America ABL800 Flex.

### Immunological collection and analysis

Blood was collected at baseline, at 0:38, 2:38, 4:38 and 8:38 (hrs:mins or at the end of the study if terminated early. Both heparinized plasma and ethylenediamine tetraacetic acid (EDTA) plasma were collected for manual complete blood counts (CBC), cytometric analysis of leukocytes, assay of biomarkers and NETs.

Manual CBC: Blood smears were prepared using 4–6 uL of well mixed EDTA blood and manual wedge technique^28^. Slides were fixed and stained using a differential stain kit (Part 9112b, Newcomer Supply, Middleton, WI). Slides were evaluated using a Zeiss Univision 200 microscope (Zeiss, Oberkochen, Germany) through a 40 × oil immersion lens. Three sets of 100 cells were evaluated and characterized into lymphocyte, neutrophil, monocyte and eosinophil and all others classified as “other.” Neutrophils were further classified into mature and immature types. A Leuko-TIC® (Medix BLT4013, Chicago, IL, USA) was used to determine leukocyte counts per volume of blood. 20 uL of blood was added to the leukotic tube for a 1:20 dilution and counted using a hemacytometer. Blood counts are expressed both as % of total (differential) and concentration in blood.

Cytometric Analysis: Samples of peripheral blood were obtained via catheter and immediately placed on ice. Once cooled, whole blood samples were incubated with fluorescein isothiocyanate (FITC)-conjugated anti-porcine CD11R3 (clone: 2F4/11, Bio-Rad Laboratories, Ann Arbor, MI) and phycoerythrin (PE)-conjugated CD172a (clone: BL1H7, Bio-Rad Laboratories, Ann Arbor, MI) for 30 min. Samples were post lysed and fixed using BD Biosciences FACS™ lysing solution (BD Biosciences, Franklin Lakes, NJ). This served a three-fold purpose: it fixed the antibody-stained leukocytes for delayed flow cytometry analysis, it destroyed remaining red blood cells, eliminating them from the analysis, and it separated leukocyte populations on the flow cytometer’s forward and side scatter plots. Data were collected using the ThermoFisher Attune Nxt acoustic focusing cytometer (ThermoFischer, Waltham, MA), and further analyzed using FlowJo software (FlowJo, Ashland, OR). Neutrophils^29,30^ and monocytes^31,32^ mobilize intracellular stores of CD11b (human equivalent to porcine CD11R3) to the cell surface as they become (primed) activated, allowing a real-time measurement of systemic acute neutrophil (priming) and monocyte activation. CD172a level is measured and then analyzed using a scatter plot method to identify neutrophils and monocytes. A typical gating strategy for cytometric analysis is explained in Supplementary Fig. 1.

Double Stranded DNA (dsDNA) was measured using PicoGreen (P7581, ThermoFisher Scientific, Waltham, MA). Plasma was diluted 1:10 and compared to a low range (1–25 ng/mL) standard curve generated with calf thymus DNA in TE buffer (10 mM Tris–HCl, 1 mM EDTA, pH 7.5). 100 uL diluted plasma or standard was combined with 100 uL of 1:400 PicoGreen and incubated for 5 min at room temperature. Fluorescence was measured using excitation ~ 480 nm, emission ~ 520 nm at a custom set PMT value. Plate background was read to check for autofluorescence prior to PicoGreen addition and found to be negligible.

Citrullinated histone H3 was assayed by ELISA using a commercially available kit (501620, Cayman Chemical, Ann Arbor, MI) developed for human and mouse and expected to react with all mammals. Samples were diluted 1:2 prior to assay per manufacturer’s instructions.

### Histology

At the end of the study, the heart was flushed with cold 10:1 heparinized NS (1L NS and 2L NS, respectively) via aortic cross clamp and cannulation and bilateral carotid cannulation, respectively, followed by the same volume of 10% formalin. Tissues were removed and stored in formalin for processing. After overnight fixation in 4% formalin (w/v) at 4 °C, a brain matrix was used to cut the cerebrum into 5 mm coronal slices and the cerebellum into 5 mm sagittal slices. The free wall of the left ventricle and right ventricle of the heart were sampled. The above specimens were immersed in PBS-buffered 30% sucrose at 4 °C for 5–7 days until they sank. All brain specimens were frozen rapidly in dry iced 2-methylbutane and stored at − 80 °C. The brain specimens were cut into 20 µm sections by cryo-sectioning with OCT embedding. The brain sections were stained with cresyl violet (AC229630050, ThermoFisher Scientific, Waltham, MA). The heart tissues were paraffin embedded and sectioned at 5 µm. The myocardial sections were stained with H&E (SH30-500 and 314-660, respectively, ThermoFisher Scientific, Waltham, MA).

Degenerated neurons in cresyl violet-stained brain sections show condensed, stained, shrunk, and angular cell bodies, while the normal neurons show light stained, large cell bodies with nucleoli. The neuronal degeneration was scored based on the percentage of degenerated neurons. Total score ranged from 0 to 4, where 0 = 0–5%;1 = 6–25%;2 = 26–50%; 3 = 51–75%; and 4 =  > 75%.

Myofiber degeneration and myocardial hemorrhage were assessed on H&E-stained ventricle sections. The absence of degeneration was scored as 0. Mild degeneration (1 +) was classified as single to multiple foci of vacuolated, shrunken, fragmented, or hypereosinophilic cells. Moderate degeneration (2 +) was classified as more frequent multifocal foci of degeneration as described above, and severe degeneration (3 +) was defined as coalescing to regionally extensive involvement. Hemorrhage was graded on a similar severity scale. The absence of hemorrhage was scored as 0. Mild focal to multifocal hemorrhage was graded as a (1 +), moderate multifocal to regionally extensive hemorrhage was graded as (2 +), and more extensive hemorrhage involving large portions of the section was graded as (3 +).

To detect the formation of NETs, immunofluorescence staining was applied. The brain or heart sections were incubated at 4 °C overnight with the following primary antibodies: mouse anti-citrullinated Histone H3 antibody (17939, Cayman Chemical, Ann Arbor, MI) and rabbit anti-von Willebrand factor antibody (NB600586, NOVUS BIOLOGICALS, Centennial, CO). Fluorescent-labeled secondary antibodies including goat Anti Rabbit IgG ALEXA FLUOR 594 (A11037, ThermoFisher Scientific, Waltham, MA), goat anti-mouse IgG AFP 488 (PIA32723, ThermoFisher Scientific, Waltham, MA) and DAPI counterstaining were used for visualization. Five views under a 20 × objective lens were randomly selected and imaged with Leica LAS X software. Fiji Image J software performed automated quantification of NETs formation as a percentage of citrullinated H3 in von Willebrand factor positive vasculature.

Cardiac and neuronal histopathology findings were compared to naïve tissue (n = 8) prepared and assessed with the same protocol of the experimental animals as described above.

### Outcomes

Primary outcomes were recovery of cardiac function at 8 hours after ECPR initiation and maximum neurologic recovery as measured by SSEPs. Cardiac resuscitability was scored via cardiac ECHO from 0 to 6 on the basis of defibrillation (Yes =1 or No =0), ROSB (Yes = 1 or No = 0), weanability from ECPR (Yes =1 or No =0), and left ventricular ejection fraction (LVEF) after weaning (Normal or mildly depressed (LVEF ≥50%) =3, Moderately depressed (LVEF 30–50%) =2, Severely depressed (LVEF ≤30%) =1). A score of 0 reflected asystole. ROSB was defined as sustained evidence of organized ventricular contractions based on transthoracic echocardiography (TTE). Weaning was determined successful if a stable MAP greater than 65 mmHg could be maintained with ECPR flow of less than 0.5 L/min. Neurologic recovery was measured as the maximum percent recovery of the porcine equivalent of the N20 neurocortical SSEP wave amplitude relative to pre-arrest baseline. Secondary outcomes include eight-hour lactate clearance (LaCl) [LaCl = *(peak lactate concentration* − *final lactate concentration) / (peak lactate concentration* × *100)].* Statistical significance was set at a *p*-value of < 0.05.

### Statistical analysis

Our power analysis for this aim is based on primary outcomes related to recovery of cardiac function (CRS range 0–6) and recovery of cortical brain function (% recovery SSEP N20 peak, range 0–20%). Based on our previous research, 12 subjects per group will provide power > 0.80 to detect a between group difference of 1.9 points on the CR score.

Demographic and clinical parameters were presented as mean ± standard deviation (SD), median [interquartile range (IQR)], or number of observations (percentage) for categorical variables. Independent samples or mixed model ANOVA were used (interaction over time) with a Tukey correction for multiple comparisons. Non-parametric data and histological data were analyzed using a Mann–Whitney test. Differences in the percent of SSEP amplitude recovery were compared using an ordinary one-way ANOVA test after confirming distribution normality using the D’Agostino and Pearson test. Statistical analyses were performed using GraphPad Prism software (GraphPad Software, Boston, MA) and R statistical software V.4.0.2 (R Foundation for Statistical Computing, Vienna, Austria). We did not perform imputation for missing data.

### Ethics approval and consent to participate

Animals in this experiment were treated in compliance with “The Guide for Care and Use of Laboratory Animals'' National Research Council of the National Academies 8th edition (revised 2011). Protocol #00011170 was approved on 1/17/2023 by the University of Michigan Institutional Animal Care and Use Committee.

## Results

**Figure 3:**
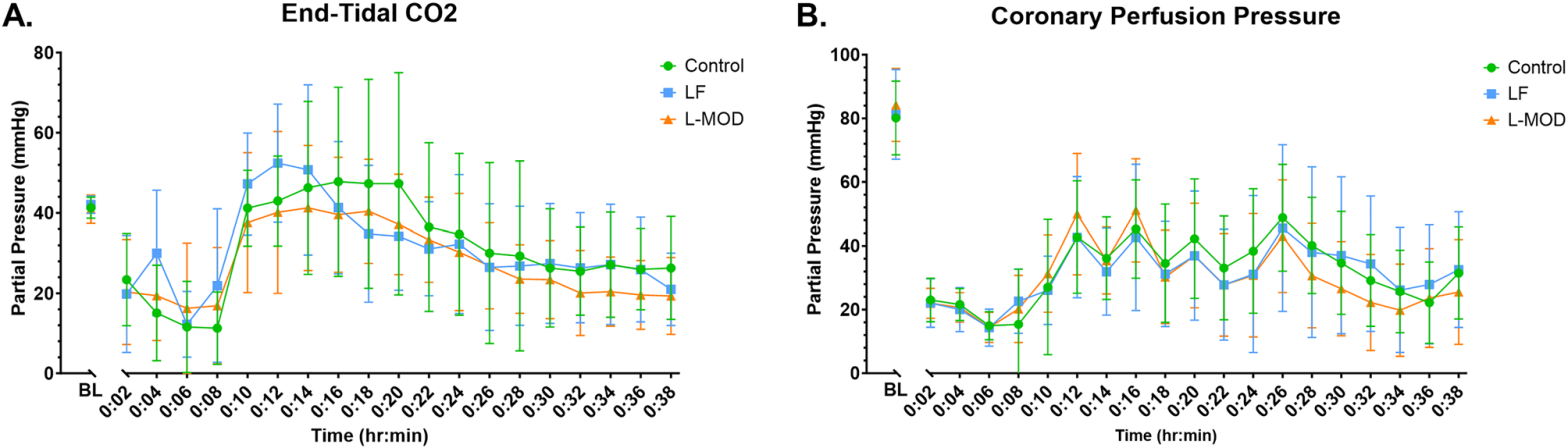
Physiologic and Hemodynamic Parameters During CPR. (**A**) End-tidal CO_2_; (**B**) Coronary perfusion pressure (CPP = aortic end-diastolic pressure—central venous pressure). Arrows represent CPR initiation and epinephrine and vasopressin doses given during resuscitation. Data are presented as mean with standard deviation. There were no statistical differences between groups. Statistical analysis was performed using repeated measures ANOVA with a significance set at *p* < 0.05 between groups.

**Figure 4:**
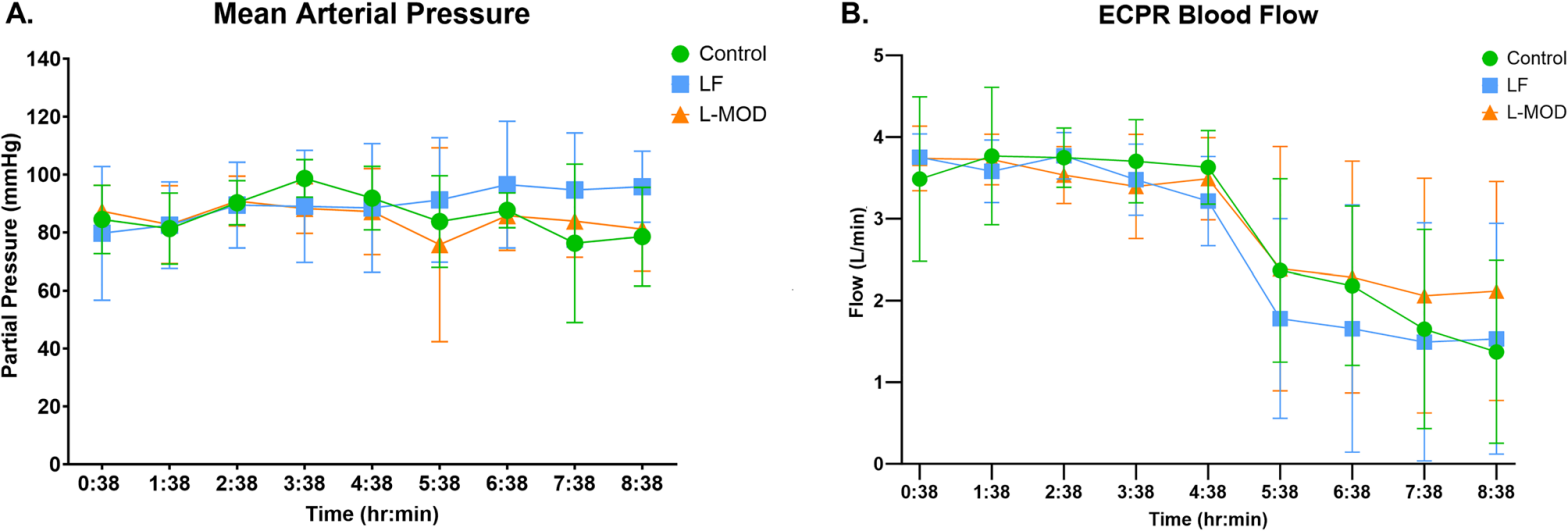
Hemodynamic Parameters During ECPR. (**A**) Mean arterial pressure; (**B**) ECPR blood flow. Data are presented as mean with standard deviation. There were no statistical differences between groups. Statistical analysis was performed using repeated measures ANOVA with a significance set at *p* < 0.05 between groups.

Among the 36 swine studied, 2 females were excluded because of CPR complications and 34 swine (Control, n = 10; LF, n = 12; L-MOD, n = 12) were analyzed. CPR complications were defined by an end-tidal CO_2_ of less than 10 mmHg for greater than 10 min. Physiologic and hemodynamic variables at baseline (Table 1), during CPR (Fig. 3), and during ECPR (Fig. 4) were not statistically different among the three groups.

**Table 1:** Baseline Physiological Parameters among Groups.

	Control n = 10	LF n = 12	L-MOD n = 12
HR (bpm)	95 (22)	100 (18)	100 (21)
MAP (mmHg)	90 (8)	88 (10)	87 (14)
CPP (mmHg)	74 (10)	72 (10)	69 (12)
pO_2_ (mmHg)	202 (30)	187 (46)	173 (55)
pCO_2_ (mmHg)	42 (3)	41 (3)	41 (5)
CVP (mmHg)	4.1 (2.3)	4.6 (2.4)	4.2 (2.2)
CO (L/min)	3.6 (0.7)	4.3 (1.1)	3.7 (0.7)

Values are mean, with standard deviation (SD) in parentheses.

Baseline physiologic variables were not statistically different between three groups. Statistical significance was set at a *p*-value of < 0.05.

### Primary and secondary outcomes

Cardiac and neurological recovery at 8 h after ECPR initiation between the three groups is shown in Table 2. There were no statistically significant differences in cardiac resuscitability score (CRS) between the groups (*p* = 0.606); 3.1 ± 2.7 in the Control group, 3.7 ± 2.6 in the LF group, and 2.6 ± 2.6 in the L-MOD group. Rates of obtaining ROSB were 70% (7/10) in the Control group, 75% (9/12) in the LF group, and 58% (7/12) in the L-MOD group. The rates of successfully weaning from ECPR were 50% (5/10) in the Control group, 58% (7/12) in the LF group, and 42% (5/12) in the L-MOD group, respectively.

**Table 2:** Primary and Secondary Outcomes.

	Control n = 10	LF n = 12	L-MOD n = 12	*p*-value
*Cardiac recovery*				
Cardiac resuscitability score	3.3 (2.7)	3.7 (2.6)	2.8 (2.6)	0.693
Success of defibrillation, n (%)^a^	7 (70)	10 (83)	8 (67)	0.623
ROSB, n (%)^b^	7 (70)	9 (75)	7 (58)	0.671
Weanability from ECPR, n (%)	6 (60)	7 (58)	6 (50)	0.875
LVEF after weaning, n (%)				
normally or mildly depressed	4 (40)	6 (50)	4 (33)	
moderately depressed	0	0	0	
severely depressed	1 (10)	0	0	
*Neurological recovery*				
% N20 amplitude return	27.9 (13.0)	36.7 (10.5)	31.2 (9.8)	0.194
N20 return > 30% of baseline, n	5	8	7	
Lactate clearance (%)	41.8 (35.7)	43.8 (37.3)	34.3 (29.5)	0.780

Values are n (%) or mean (SD). ^a^Defibrillation success was defined as return of stable supraventricular rhythm. ^b^ROSB was assessed on the basis of the evidence of heart contraction by qualitative echocardiography.

The primary cardiac recovery outcome was measured as CRS which was based on our previous publication (26). The CRS ranges from 0 to 6 based on defibrillation (Yes = 1 or No = 0); ROSB (Yes = 1 or No = 0); weanability from ECPR (Yes = 1 or No = 0); and left ventricular ejection fraction (LVEF) after weaning (Normal or mildly depressed = 3, Moderately depressed = 2, Severely depressed = 1). Defibrillation success was defined as the return of stable supraventricular rhythm. ROSB was assessed based on the evidence of heart contraction by qualitative echocardiography. Neurologic recovery was measured as the maximum percent recovery of the porcine equivalent of the N20 neurocortical SSEP wave amplitude relative to pre-arrest baseline. Statistical significance was set at a *p*-value of < 0.05.

Secondary outcomes include eight-hour lactate clearance (LaCl) [LaCl = *(peak lactate concentration—final lactate concentration) / (peak lactate concentration* × *100)].* Statistical significance was set at a *p*-value of < 0.05.

**Figure 5:**
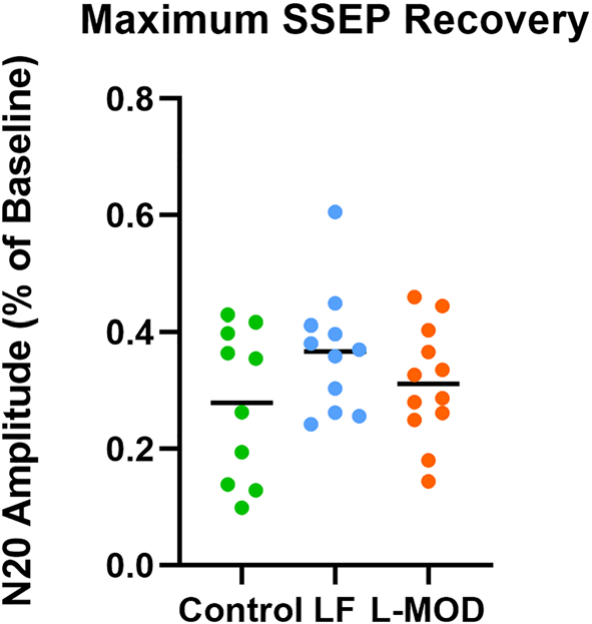
Maximum SSEP Recovery. Percent recovery from baseline of the porcine equivalent of the N20 neurological wave. The horizontal line represents the median. No statistically significant differences were observed between groups with a significance set at *p* < 0.05.

There were no significant differences in the maximum percent of cortical SSEP amplitude recovery between the groups (*p* = 0.194); 27.9 ± 13.0% in the Control group, 36.7 ± 10.5% in the LF group, and 31.2 ± 9.8% in the L-MOD group. The number of animals in each group to achieve ≥ 30% amplitude recovery of the N20 was 5 in the Control group, 8 in the LF group, and 7 in the L-MOD group (Fig. 5).

There was not a significant difference in LaCl among the groups (*p* = 0.78); Control = 41.8% (35.7%), LF = 43.8% (37.3%), and L-MOD = 34.4% (29.5%) **(**Table 2**)**.

### Histopathological analysis

**Figure 6:**
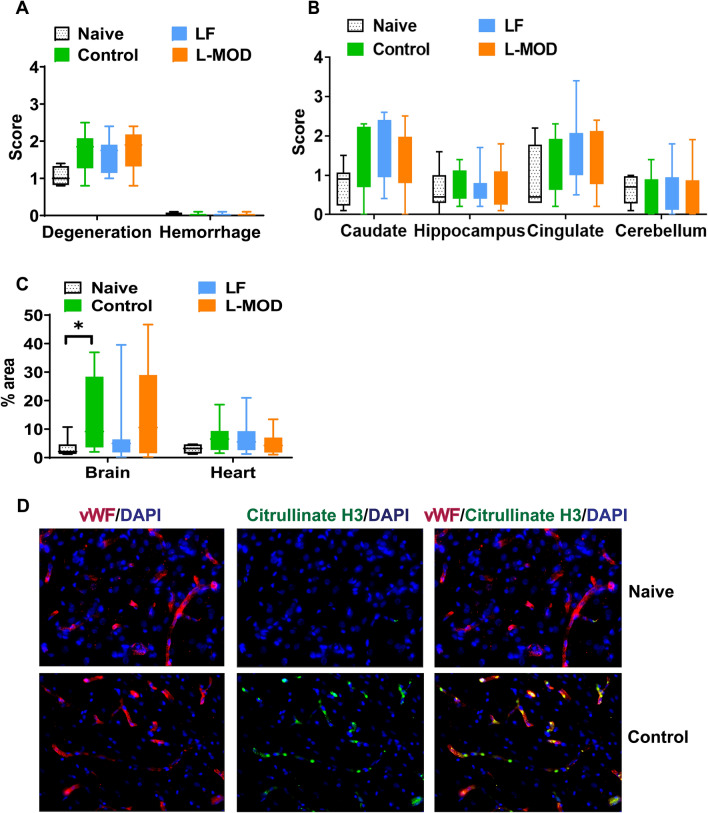
Cardiac and Neuronal Histopathology (**A**) Myocardial injuries. Myofiber degeneration and myocardial hemorrhage are shown in box and whiskers plots. Sections of ventricles were stained with H&E staining. The myofiber degeneration and myocardial hemorrhage were graded from 0 to 3. The data were analyzed with Mann–Whitney test. Naïve n = 4, Control n = 8, LF n = 12, L-MOD n = 12. (**B**) Neuronal injuries in the brain. Neuronal degeneration are shown in box and whiskers plots. Brain sections were stained with cresyl violet. The neuronal degeneration scores at caudate, hippocampus, cingulate cortex, and cerebellum were graded from 0 to 4 based on the percentage of degenerated neurons. 0: 0–5%, 1: 6%-25%, 2: 26%-50%, 3: 51%-75%, and 4: 76%-100%. The data were analyzed with Mann–Whitney test. Naïve n = 8, Control n = 8, LF n = 12, L-MOD n = 12. (**C**) Neutrophil extracellular traps (NETs) in vasculatures. The colocalization of citrullinated Histone H3 in von Willebrand Factor (vWF) was detected with immunofluorescence staining. The NETs formations were quantified with Fiji Image J as percentage of citrullinated histone H3 area in von Willebrand Factor-stained vasculature. Brain: Naïve n = 8, Control n = 8, LF n = 12, L-MOD n = 12. Heart: Naïve n = 4, Control n = 8, LF n = 12, L-MOD n = 12. The data were analyzed with Mann–Whitney test. *p < 0.05 Naïve vs. Control. (**D**) Representative images of NETs immunofluorescent staining (200x) in brain tissue. The colocalization of von Willebrand Factor (Red) and citrullinated H3 (Green) was detected with immunofluorescence. DAPI counterstaining shows nuclei (blue). Top panel: NETs in Naïve group. Bottom panel: NETs in Control group.

There were no significant differences in myocardial degeneration between the Naïve and Control groups (*p* = 0.053), between the LF and Control groups (*p* = 0.583), or between the L-MOD and Control groups (*p* = 0.865) (Fig. 6A). The myocardial hemorrhage scores were not different between Naïve versus Control (*p* > 0.99), LF versus Control (*p* > 0.99), and L-MOD versus Control groups (*p* > 0.99) (Supplementary Table 1A).

Neuronal degeneration was evaluated at four different locations in the brain. At the caudate nucleus, there were no significant differences between the groups (Naïve vs. Control, *p* = 0.0991; LF vs. Control, *p* = 0.583; L-MOD vs. Control, *p* = 0.777). Similarly, no significant differences were observed at the hippocampus (Naïve vs. Control, *p* = 0.626; LF vs. Control, *p* = 0.520; L-MOD vs. Control, *p* = 0.530), the cingulate cortex (Naïve vs. Control, *p* = 0.393; LF vs. Control, *p* = 0.314; L-MOD vs. Control, *p* = 0.482), or the cerebellum (Naïve vs. Control, *p* = 0.320; LF vs. Control, *p* = 0.435; L-MOD vs. Control, *p* = 0.834). Please refer to Fig. 6B and Supplementary Table 1B for details.

As a NETs marker, citrullinated Histone H3 was detected in the vasculatures of both the heart and the cerebral cortex (Fig. 6C**, **D). The NETs in the heart were mainly detected in small blood vessels (Supplementary Fig. 2). No significant differences were observed among the groups for Naïve versus Control (*p* = 0.109), LF versus Control (*p* = 0.851), and L-MOD versus Control (*p* = 0.384) (Supplementary Table 1C). The NETs in the brain were mainly detected in capillaries, and the Control group had significantly higher brain NETs compared to the Naïve group (*p* = 0.021). No significant differences were observed between the Control and LF groups (*p* = 0.28) or L-MOD groups (*p* = 0.955) (Supplementary Table 1D).

### Immunological analysis

**Figure 7:**
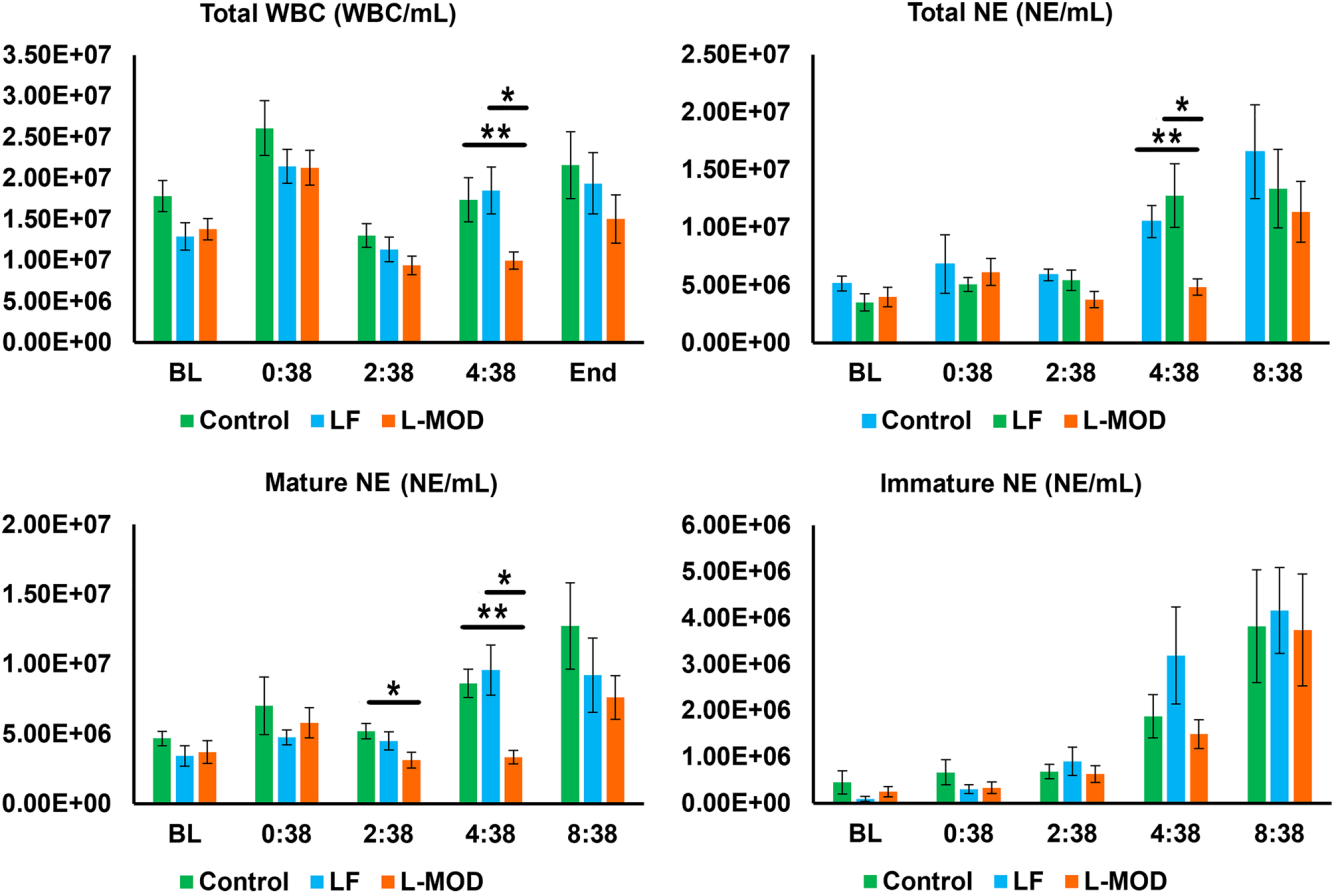
Neutrophil count throughout ECPR. Neutrophil populations between groups is shown in the figure. A statistically significant difference in total WBC was observed at time point 4:38 between the L-MOD group and both the Control and LF groups. A statistically significant difference in total neutrophil count was observed at time point 4:38 between the L-MOD group and both the Control and LF groups. A statistically significant difference in mature neutrophil count was observed at time point 2:38 between the Control and L-MOD groups and at 4:38 between the L-MOD group and both the Control and LF groups, respectively. Statistical significance was set at a *p*-value of < 0.05.

L-MOD therapy resulted in statistically reduced total WBCs, total neutrophils, and mature neutrophils at 4 h after ECPR onset (Fig. 7). Leukocyte counts were not different between Control and LF groups at any experimental timepoint.

**Figure 8:**
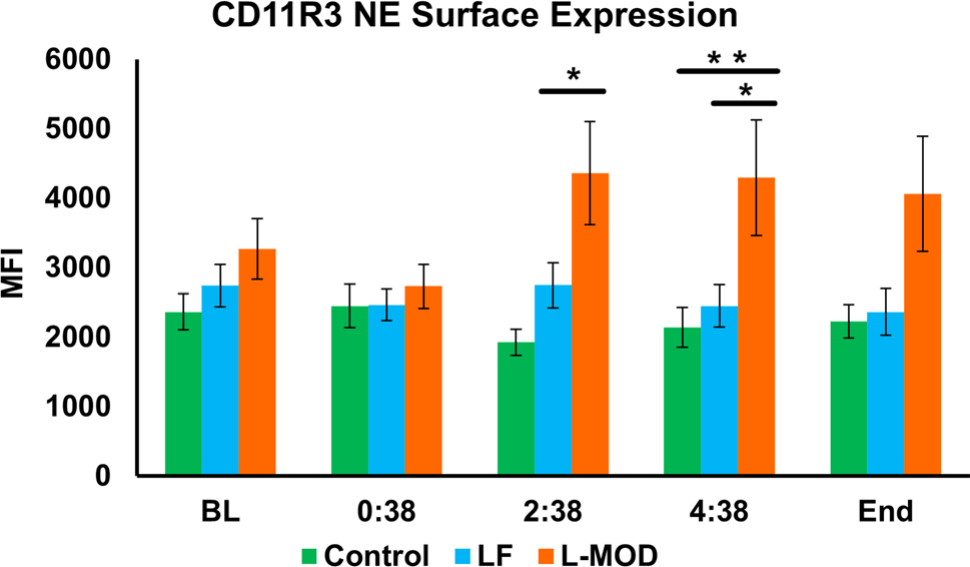
CD11R3 Neutrophil Surface Expression. There were no significant differences in CD11R3 expression among the groups at baseline, 0:38, or at the end of the study. At 2:38 the L-MOD group had a significantly higher CD11R3 NE expression than the Control group (*p* = 0.014). Additionally, at 4:38, the L-MOD group had a significantly higher CD11R3 NE expression than the Control and LF groups (*p* = 0.048 and *p* = 0.0498, respectively). Statistical significance was set at a *p*-value of < 0.05.

The MFI of CD11R3 neutrophil surface expression is shown in Fig. 8. There were no significant differences in CD11R3 expression among the groups at baseline, 0:38, or at the end of the study. At 2:38 the L-MOD group had a significantly higher CD11R3 NE expression than the Control group (*p* = 0.014). Additionally, at 4:38, the L-MOD group had a significantly higher CD11R3 NE expression than the Control and LF groups (*p* = 0.048 and *p* = 0.0498, respectively).

**Figure 9:**
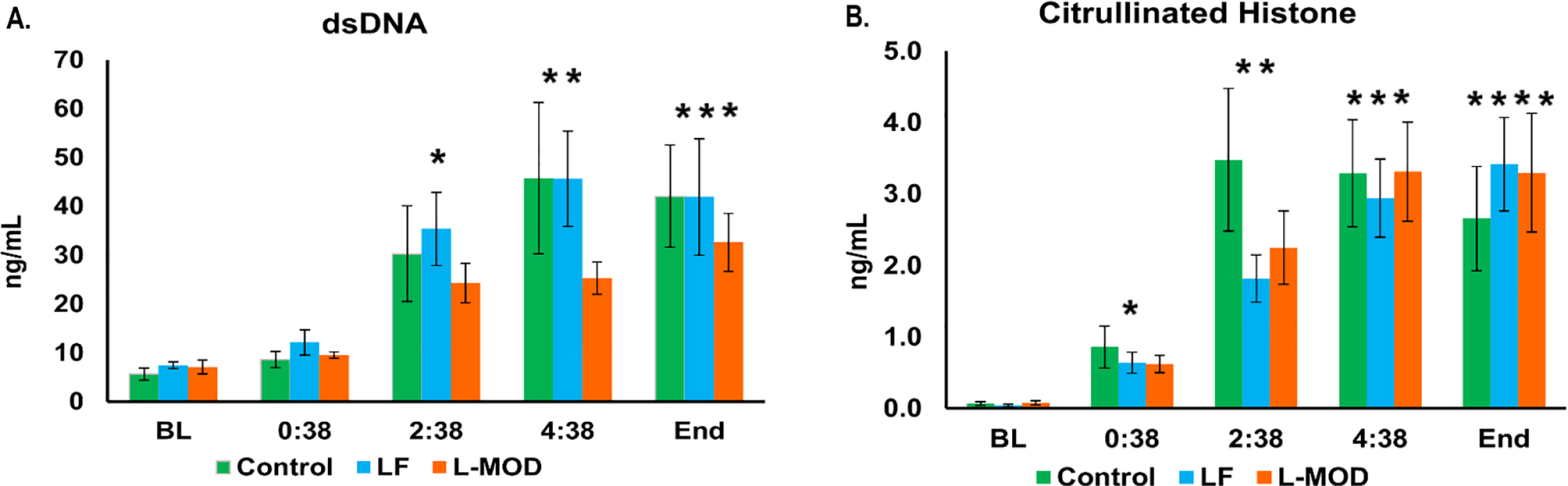
NETosis Markers. (**A**) Double-Stranded DNA (dsDNA). DsDNA was measured to serve as a surrogate for NETosis. Groups were not significantly different among each other at any time point. However, there was a significant increase in dsDNA compared to baseline for all three groups at timepoints 2:38, 4:38 and the end. (**B**) Citrullinated Histone. Citrullinated Histone H3 Elisa results are graphed above. Groups were not significantly different among each other at any time point. However, there was a significant increase in citrullinated histone compared to baseline for all three groups at timepoints 0:38, 2:38, 4:38 and the end. Statistical significance was set at a *p*-value of < 0.05.

Serum dsDNA and citrullinated histone H3 were quantified as biomarkers of NETosis (Fig. 9). There were no significant differences in the mean concentration of dsDNA among the groups at any time point throughout the study; however, there was a significant increase in dsDNA compared to baseline for all three groups at timepoints 2:38, 4:38 and the end (*p* < 0.05) (Fig. 9A). There were no significant differences in the mean concentration of citrullinated histone among the groups at any time point; however, there was a significant increase in citrullinated histone compared to baseline for all three groups at timepoints 0:38, 2:38, 4:38 and the end (Fig. 9B).

## Discussion

In this model of prolonged cardiac arrest treated with ECPR, we observed elevated biomarkers of NETosis, microvascular NET formation in the heart and brain as well as impaired recovery of both cardiac and neurologic function. However, LF and L-MOD therapies did not have a significant impact on NETosis or recovery of heart and brain function. These results suggest that these modalities as applied in this study are not an effective strategy to reduce no-reflow caused by NETosis.

### No-reflow phenomenon in cardiac arrest

Since the first description of the “no-reflow” phenomenon by Ames in 1968^33^, subsequent research has provided compelling evidence that no-reflow occurs after focal and global ischemia of sufficient duration in every organ and species tested. However, there is conflicting evidence regarding the causal mechanisms. The best available evidence implicates microvascular thrombosis, leukocyte adhesion, and neutrophil extracellular traps (NETs) as major contributors^8,34–38^. Downstream inflammatory and injury cascades triggered by these mechanisms may also cause endothelial cell edema and pericyte-mediated capillary constriction^39^.

### Leukocyte adhesion and NET formation during prolonged cardiac arrest

Initial shedding of the microvascular endothelial glycocalyx during ischemia and reperfusion is thought to expose adhesion molecules that cause leukocyte adhesion^40^. Adherent neutrophils (NE) and monocytes in capillaries and post-capillary venules can mechanically disrupt flow by themselves or do so by forming neutrophil extracellular traps^8,34^. They can also release proteases such as elastase that amplify damage to the endothelial glycocalyx, and superoxide that causes local free radical injury, all of which contributes to a feed-forward injury pathway amplifying leukocyte adhesion^41^. In addition, oxidative injury can cause capillary pericyte constriction, resulting in local obstruction and shunting of blood flow^42,43^. Neutrophil extracellular traps (NETs) are formed when activated neutrophils undergo apoptosis and extrude their nuclear contents forming an intravascular scaffold made of nuclear DNA and hypercitrullinated histones in a process that has been termed “NETosis”^44,45^. Although the primary function is thought to be antibacterial, there is a compelling body of evidence that NETs provide a unique link between inflammation and thrombosis by providing a scaffold and stimulus for intravascular thrombus formation through platelet and RBC aggregation and local concentration of fibrinogen, fibronectin, and von Willebrand factor^46^. The negatively charged DNA backbone of NETs activates factor XII, while positively charged histones activate platelets via TLR2 and TLR4 signaling. In addition, neutrophil elastase and cathepsin G released during NETosis degrade tissue factor pathway inhibitors. Hirose et al. reported that blood smears obtained at time of ICU admission from 5 out of 8 post-CA patients were immunolabeled for either circulating NETs or citrullinated histones^47^. Although this result is far from establishing a role for NETs in post-CA no-reflow, it does demonstrate that the pathologic process of NET formation is activated in this patient population. The intriguing function of NETs amplifies the importance of studying the role of activated neutrophils as a contributor to no-flow after prolonged CA.

### Strategies to mitigate NETosis

Effective and safe strategies to mitigate the leukocyte-mediated injury mechanism have been elusive. ECPR provides a unique opportunity to use in-circuit devices to modulate leukocyte activation starting at the time of initial reperfusion, when impact of such therapy may be greatest. Leukocyte filters (40 µm) in the arterial (re-infusion) line of the cardiopulmonary bypass (CPB) and ECMO circuits have been developed and evaluated for prophylactic use to reduce the number of activated circulating neutrophils in preclinical animal models and clinical studies^48^. Data in humans are controversial and conflicting as some studies have demonstrated a significant decrease in leukocyte counts associated with less myocardial damage^49–51^, improvement of left ventricular function^52^, decreased lung injury^48,50,53–56^, and mild renal protection benefits^57^. Other studies have shown no clinical benefit during the use of leukocyte filters in the arterial line^18,58–60^. Acknowledgement of “filter exhaustion,” a progressive decrease in leukocyte reduction efficiency during CPB, has been repeatedly observed during experimental evaluation of leukocyte filtration^14,53,61,62^. In this study the Leukoguard blood filter proved ineffective in reducing the number of circulating leukocytes and therefore no benefit was observed over the Control group. This observation is shared by others studying the cardiopulmonary bypass model and ex vivo perfusion circuits^63,64^.

Co-investigator Humes has developed a novel alternative approach utilizing an innovative biomimetic membrane called the Leukocyte Modulation Device (L-MOD). The L-MOD is a biomimetic membrane device consisting of specialized fiber membranes within a polycarbonate housing (Fig. 2B). These fibers have a unique ability to temporarily bind and sequester certain leukocytes (specifically neutrophils and monocytes). When used in combination with the pharmacologic effects of citrate regional anticoagulation, bound activated leukocytes are immunomodulated and released back to the circulation, which alters the phenotype of circulating leukocytes and attenuates systemic inflammation^20,53,62,65^. Early pre-clinical and clinical results suggest that the L-MOD ameliorates the multi-organ dysfunction effects of systemic inflammatory response syndrome (SIRS) and has an impact on the mortality rate of multi-organ failure in ICU patients^22,66^. Rather than target soluble biomarkers of inflammation, L-MOD therapy focuses on modulating effector cells.

In this study, CD11R3 expression levels, the porcine equivalent of CD11b in humans, were highly variable at baseline and throughout the study among individual animals in the Control and LF groups. CD11R3 expression has been demonstrated to increase post insult in pig models (CPB, sepsis) and expression levels shown to decrease upon intervention with devices such as the L-MOD^21,67^. This was not observed in our ECPR model. For the L-MOD group, CD11R3 was higher post intervention compared to baseline. One hypothesis as to why an increase in CD11R3 was not observed in circulating NE in the Control and LF groups was that they were actively being recruited to tissues^21^.

The serum dsDNA, serum citrullinated histone and citrullinated histone immunohistochemistry in the brain and heart were not changed by the LF or L-MOD therapy. Although no significant differences were observed between groups, the L-MOD group trended lower, thus the treatment may have impacted NETosis. This suggests that circulating dsDNA and citrullinated histone may be reliable biomarkers for tissue NETosis. DsDNA has been used by others as a surrogate measurement for NETosis^68,69^.

### Study limitations

This study has several limitations. First, the relatively short period of observation after CA limits the assessment of any treatment effect on long-term recovery of organ function. Second, we did not directly measure microvascular no-reflow. Therefore, the impact of the study interventions on the no-reflow phenomenon was not directly assessed. Third, the burden of microvascular NET formation may not have been severe enough in this model for the therapies to have a detectable benefit. Finally, there does not currently exist an animal model that identically represents the clinical setting this study attempted to replicate. Therefore, the impact of LF or L-MOD therapy on NETtosis and no-reflow could be different under various clinical conditions in which ECPR is used to treat prolonged cardiac arrest.

## Conclusion

In this swine model of prolonged cardiac arrest treated with ECPR, the use of LF or L-MOD therapy in tandem with ECPR did not reduce NETosis or improve recovery of cardiac and neurologic function. The causal relationship between microvascular NETosis, no-reflow, and recovery of organ function after prolonged cardiac arrest treated with ECPR requires further investigation.

### Supplementary Information

Supplementary Information.

## Data Availability

The datasets used and/or analyzed during the current study are available from the corresponding author on reasonable request.

## Abbreviations

ACDA: Acid citrate dextrose anticoagulant
BL: Baseline
Ca: Calcium
CA: Cardiac arrest
CO: Cardiac output
CPP: Coronary perfusion pressure
CPR: Cardiopulmonary resuscitation
CRS: Cardiac resuscitability score
CVP: Central venous pressure
DP: Dialysis pump
dsDNA: Double-stranded deoxyribonucleic acid
ECMO: Extracorporeal membrane oxygenation
ECPR: Extracorporeal cardiopulmonary resuscitation
HR: Heart rate
iCa: Ionized calcium
LF: Leukocyte filter
L-MOD: Leukocyte modulation device
LVEF: Left ventricular ejection fraction
MAP: Mean arterial pressure
MFI: Mean fluorescence intensity
NE: Neutrophils
NETs: Neutrophil extracellular traps
RCA: Regional citrate anticoagulation
ROSB: Return of spontaneous beat
SCD: Selective cytopheretic device
SSEP: Somatosensory evoked potentials
TTE: Transthoracic echocardiography
VF: Ventricular fibrillation
vWF: Von Willebrand factor
WBC: White blood cell

## References

[1] Yannopoulos D, Bartos J, Raveendran G, Walser E, Connett J, Murray TA (2020). Advanced reperfusion strategies for patients with out-of-hospital cardiac arrest and refractory ventricular fibrillation (ARREST): A phase 2, single centre, open-label, randomised controlled trial. Lancet Lond. Engl..

[2] Hsu CH, Meurer WJ, Domeier R, Fowler J, Whitmore SP, Bassin BS (2021). Extracorporeal cardiopulmonary resuscitation for refractory out-of-hospital cardiac arrest (EROCA): Results of a randomized feasibility trial of expedited out-of-hospital transport. Ann. Emerg. Med..

[3] Belohlavek J, Smalcova J, Rob D, Franek O, Smid O, Pokorna M (2022). Effect of intra-arrest transport, extracorporeal cardiopulmonary resuscitation, and immediate invasive assessment and treatment on functional neurologic outcome in refractory out-of-hospital cardiac arrest: A randomized clinical trial. JAMA.

[4] Suverein MM, Delnoij TSR, Lorusso R, Brandon Bravo Bruinsma GJ, Otterspoor L, Elzo Kraemer CV (2023). Early extracorporeal CPR for refractory out-of-hospital cardiac arrest. N .Engl. J. Med..

[5] Zhang Y, Peng R, Pei S, Gao S, Sun Y, Cheng G (2023). Neutrophil extracellular traps are increased after extracorporeal membrane oxygenation support initiation and present in thrombus: A preclinical study using sheep as an animal model. Thromb. Res..

[6] Mauracher LM, Buchtele N, Schörgenhofer C, Weiser C, Herkner H, Merrelaar A (2019). Increased citrullinated histone H3 levels in the early post-resuscitative period are associated with poor neurologic function in cardiac arrest survivors-a prospective observational study. J. Clin. Med..

[7] Li P, Liang S, Wang L, Guan X, Wang J, Gong P (2023). Predictive value of neutrophil extracellular trap components for 28-day all-cause mortality in patients with cardiac arrest: A pilot observational study. Shock.

[8] Ge L, Zhou X, Ji WJ, Lu RY, Zhang Y, Zhang YD (2015). Neutrophil extracellular traps in ischemia-reperfusion injury-induced myocardial no-reflow: Therapeutic potential of DNase-based reperfusion strategy. Am. J. Physiol. Heart Circ. Physiol..

[9] Neumar RW, Nolan JP, Adrie C, Aibiki M, Berg RA, Böttiger BW (2008). Post-cardiac arrest syndrome: epidemiology, pathophysiology, treatment, and prognostication. A consensus statement from the International Liaison Committee on Resuscitation (American Heart Association, Australian and New Zealand Council on Resuscitation, European Resuscitation Council, Heart and Stroke Foundation of Canada, InterAmerican Heart Foundation, Resuscitation Council of Asia, and the Resuscitation Council of Southern Africa); the American Heart Association Emergency Cardiovascular Care Committee; the Council on Cardiovascular Surgery and Anesthesia; the Council on Cardiopulmonary, Perioperative, and Critical Care; the Council on Clinical Cardiology; and the Stroke Council. Circulation..

[10] Adrie C, Adib-Conquy M, Laurent I, Monchi M, Vinsonneau C, Fitting C (2002). Successful cardiopulmonary resuscitation after cardiac arrest as a “sepsis-like” syndrome. Circulation.

[11] Langeland H, Damås JK, Mollnes TE, Ludviksen JK, Ueland T, Michelsen AE (2022). The inflammatory response is related to circulatory failure after out-of-hospital cardiac arrest: A prospective cohort study. Resuscitation.

[12] Millar JE, Fanning JP, McDonald CI, McAuley DF, Fraser JF (2016). The inflammatory response to extracorporeal membrane oxygenation (ECMO): A review of the pathophysiology. Crit. Care Lond. Engl..

[13] Yao Y, Kang H, Cheng Y, Su X, Wang B (2023). Inflammatory progression in patients undergoing extracorporeal membrane oxygenation. Curr. Mol. Med..

[14] Warren O, Alexiou C, Massey R, Leff D, Purkayastha S, Kinross J (2007). The effects of various leukocyte filtration strategies in cardiac surgery. Eur. J. Cardio-Thorac. Surg. Off. J. Eur. Assoc. Cardio-Thorac. Surg..

[15] Rimpiläinen J, Pokela M, Kiviluoma K, Anttila V, Vainionpää V, Hirvonen J (2000). Leukocyte filtration improves brain protection after a prolonged period of hypothermic circulatory arrest: A study in a chronic porcine model. J. Thorac. Cardiovasc. Surg..

[16] Alaoja H, Niemelä E, Anttila V, Dahlbacka S, Mäkelä J, Kiviluoma K (2006). Leukocyte filtration to decrease the number of adherent leukocytes in the cerebral microcirculation after a period of deep hypothermic circulatory arrest. J. Thorac. Cardiovasc. Surg..

[17] Boodram S, Evans E (2008). Use of leukocyte-depleting filters during cardiac surgery with cardiopulmonary bypass: A review. J. Extra Corpor. Technol..

[18] Sahlman A, Ahonen J, Salo JA, Rämö OJ (2001). No impact of a leucocyte depleting arterial line filter on patient recovery after cardiopulmonary bypass. Acta Anaesthesiol. Scand..

[19] Salamonsen RF, Anderson J, Anderson M, Bailey M, Magrin G, Rosenfeldt F (2005). Total leukocyte control for elective coronary bypass surgery does not improve short-term outcome. Ann. Thorac. Surg..

[20] Pino CJ, Lou L, Smith PL, Ding F, Pagani FD, Buffington DA (2012). A selective cytopheretic inhibitory device for use during cardiopulmonary bypass surgery. Perfusion.

[21] Johnston KA, Westover AJ, Rojas-Pena A, Haft JW, Toomasian JM, Johnson T (2019). Novel leukocyte modulator device reduces the inflammatory response to cardiopulmonary bypass. ASAIO J. Am. Soc. Artif. Intern. Organs.

[22] Ding F, Yevzlin AS, Xu ZY, Zhou Y, Xie QH, Liu JF (2011). The effects of a novel therapeutic device on acute kidney injury outcomes in the intensive care unit: A pilot study. ASAIO J. Am. Soc. Artif. Intern. Organs.

[23] Tumlin JA, Chawla L, Tolwani AJ, Mehta R, Dillon J, Finkel KW (2013). The effect of the selective cytopheretic device on acute kidney injury outcomes in the intensive care unit: A multicenter pilot study. Semin. Dial..

[24] Tumlin JA, Galphin CM, Tolwani AJ, Chan MR, Vijayan A, Finkel K (2015). A multi-center, randomized, controlled, pivotal study to assess the safety and efficacy of a selective cytopheretic device in patients with acute kidney injury. PloS One.

[25] VanZalen JJ, Harvey S, Hála P, Phillips A, Nakashima T, Gok E (2023). Therapeutic effect of argatroban during cardiopulmonary resuscitation and streptokinase during extracorporeal cardiopulmonary resuscitation in a porcine model of prolonged cardiac arrest. Crit. Care Explor..

[26] Spinelli E, Davis RP, Ren X, Sheth PS, Tooley TR, Iyengar A (2016). Thrombolytic-enhanced extracorporeal cardiopulmonary resuscitation after prolonged cardiac arrest. Crit. Care Med..

[27] Yessayan LT, Neyra JA, Westover AJ, Szamosfalvi B, Humes HD (2022). Extracorporeal immunomodulation treatment and clinical outcomes in ICU COVID-19 patients. Crit. Care Explor..

[28] Rosenthal, D. S. UpToDate. [cited 2024 Jan 20]. Evaluation of the peripheral blood smear - UpToDate. Available from: https://www.uptodate.com/contents/evaluation-of-the-peripheral-blood-smear

[29] Hamblin A, Taylor M, Bernhagen J, Shakoor Z, Mayall S, Noble G (1992). A method of preparing blood leucocytes for flow cytometry which prevents upregulation of leucocyte integrins. J. Immunol. Methods.

[30] Finn A, Rebuck N (1994). Measurement of adhesion molecule expression on neutrophils and fixation. J. Immunol. Methods.

[31] Lundahl J, Halldén G, Sköld CM (1996). Human blood monocytes, but not alveolar macrophages, reveal increased CD11b/CD18 expression and adhesion properties upon receptor-dependent activation. Eur. Respir. J..

[32] Fontes ML, Mathew JP, Rinder HM, Zelterman D, Smith BR, Rinder CS (2005). Atrial fibrillation after cardiac surgery/cardiopulmonary bypass is associated with monocyte activation. Anesth. Analg..

[33] Ames A, Wright RL, Kowada M, Thurston JM, Majno G (1968). Cerebral ischemia. II. The no-reflow phenomenon. Am. J. Pathol..

[34] del Zoppo GJ, Schmid-Schönbein GW, Mori E, Copeland BR, Chang CM (1991). Polymorphonuclear leukocytes occlude capillaries following middle cerebral artery occlusion and reperfusion in baboons. Stroke.

[35] Fischer M, Hossmann KA (1995). No-reflow after cardiac arrest. Intensive Care Med..

[36] Fischer M, Böttiger BW, Popov-Cenic S, Hossmann KA (1996). Thrombolysis using plasminogen activator and heparin reduces cerebral no-reflow after resuscitation from cardiac arrest: an experimental study in the cat. Intensive Care Med..

[37] Jordan JE, Zhao ZQ, Vinten-Johansen J (1999). The role of neutrophils in myocardial ischemia-reperfusion injury. Cardiovasc. Res..

[38] Mori E, del Zoppo GJ, Chambers JD, Copeland BR, Arfors KE (1992). Inhibition of polymorphonuclear leukocyte adherence suppresses no-reflow after focal cerebral ischemia in baboons. Stroke.

[39] Dalkara T, Alarcon-Martinez L (2015). Cerebral microvascular pericytes and neurogliovascular signaling in health and disease. Brain Res..

[40] Chappell D, Westphal M, Jacob M (2009). The impact of the glycocalyx on microcirculatory oxygen distribution in critical illness. Curr. Opin. Anaesthesiol..

[41] Becker BF, Jacob M, Leipert S, Salmon AHJ, Chappell D (2015). Degradation of the endothelial glycocalyx in clinical settings: Searching for the sheddases. Br. J. Clin. Pharmacol..

[42] O’Farrell FM, Attwell D (2014). A role for pericytes in coronary no-reflow. Nat. Rev. Cardiol..

[43] Yemisci M, Gursoy-Ozdemir Y, Vural A, Can A, Topalkara K, Dalkara T (2009). Pericyte contraction induced by oxidative-nitrative stress impairs capillary reflow despite successful opening of an occluded cerebral artery. Nat. Med..

[44] Fuchs TA, Abed U, Goosmann C, Hurwitz R, Schulze I, Wahn V (2007). Novel cell death program leads to neutrophil extracellular traps. J. Cell Biol..

[45] Sørensen OE, Borregaard N (2016). Neutrophil extracellular traps - The dark side of neutrophils. J. Clin. Invest..

[46] Gould TJ, Lysov Z, Liaw PC (2015). Extracellular DNA and histones: Double-edged swords in immunothrombosis. J. Thromb. Haemost. JTH.

[47] Hirose T, Hamaguchi S, Matsumoto N, Irisawa T, Seki M, Tasaki O (2014). Presence of neutrophil extracellular traps and citrullinated histone H3 in the bloodstream of critically ill patients. PloS One.

[48] Karaiskos TE, Palatianos GM, Triantafillou CD, Kantidakis GH, Astras GM, Papadakis EG (2004). Clinical effectiveness of leukocyte filtration during cardiopulmonary bypass in patients with chronic obstructive pulmonary disease. Ann. Thorac. Surg..

[49] Matheis G, Scholz M, Gerber J, Abdel-Rahman U, Wimmer-Greinecker G, Moritz A (2001). Leukocyte filtration in the early reperfusion phase on cardiopulmonary bypass reduces myocardial injury. Perfusion.

[50] Chen YF, Tsai WC, Lin CC, Tsai LY, Lee CS, Huang CH (2004). Effect of leukocyte depletion on endothelial cell activation and transendothelial migration of leukocytes during cardiopulmonary bypass. Ann. Thorac. Surg..

[51] Sutton SW, Patel AN, Chase VA, Schmidt LA, Hunley EK, Yancey LW (2005). Clinical benefits of continuous leukocyte filtration during cardiopulmonary bypass in patients undergoing valvular repair or replacement. Perfusion.

[52] Di Salvo C, Louca LL, Pattichis K, Hooper J, Walesby RK (1996). Does activated neutrophil depletion on bypass by leukocyte filtration reduce myocardial damage? A preliminary report. J. Cardiovasc. Surg. (Torino).

[53] Alexiou C, Tang AAT, Sheppard SV, Smith DC, Gibbs R, Livesey SA (2004). The effect of leucodepletion on leucocyte activation, pulmonary inflammation and respiratory index in surgery for coronary revascularisation: A prospective randomised study. Eur. J. Cardio-Thorac. Surg. Off. J. Eur. Assoc. Cardio-Thorac. Surg..

[54] Alexiou C, Tang ATM, Sheppard SV, Haw MP, Gibbs R, Smith DC (2004). A prospective randomized study to evaluate the effect of leukodepletion on the rate of alveolar production of exhaled nitric oxide during cardiopulmonary bypass. Ann. Thorac. Surg..

[55] Sheppard SV, Gibbs RV, Smith DC (2004). Does the use of leucocyte depletion during cardiopulmonary bypass affect exhaled nitric oxide production?. Perfusion.

[56] Sheppard SV, Gibbs RV, Smith DC (2004). Does leucocyte depletion during cardiopulmonary bypass improve oxygenation indices in patients with mild lung dysfunction?. Br. J. Anaesth..

[57] Tang ATM, Alexiou C, Hsu J, Sheppard SV, Haw MP, Ohri SK (2002). Leukodepletion reduces renal injury in coronary revascularization: A prospective randomized study. Ann. Thorac. Surg..

[58] Mihaljevic T, Tönz M, von Segesser LK, Pasic M, Grob P, Fehr J (1995). The influence of leukocyte filtration during cardiopulmonary bypass on postoperative lung function. A clinical study. J. Thorac. Cardiovasc. Surg..

[59] Fabbri A, Manfredi J, Piccin C, Soffiati G, Carta MR, Gasparotto E (2001). Systemic leukocyte filtration during cardiopulmonary bypass. Perfusion.

[60] Leal-Noval SR, Amaya R, Herruzo A, Hernández A, Ordóñez A, Marín-Niebla A (2005). Effects of a leukocyte depleting arterial line filter on perioperative morbidity in patients undergoing cardiac surgery: A controlled randomized trial. Ann. Thorac. Surg..

[61] Hurst T, Johnson D, Cujec B, Thomson D, Mycyk T, Burbridge B (1997). Depletion of activated neutrophils by a filter during cardiac valve surgery. Can. J. Anaesth. J. Can. Anesth..

[62] Smit JJ, de Vries AJ, Gu YJ, van Oeveren W (1999). Efficiency and safety of leukocyte filtration during cardiopulmonary bypass for cardiac surgery. Transfus. Sci..

[63] Fahradyan V, Annunziata MJ, Said S, Rao M, Shah H, Ordenana C (2020). Leukoreduction in ex vivo perfusion circuits: Comparison of leukocyte depletion efficiency with leukocyte filters. Perfusion.

[64] Koskenkari JK, Rimpiläinen J, Ohman H, Surcel HM, Vainionpää V, Biancari F (2006). Leukocyte filter enhances neutrophil activation during combined aortic valve and coronary artery bypass surgery. Heart Surg. Forum.

[65] Ding F, Song JH, Jung JY, Lou L, Wang M, Charles L (2011). A biomimetic membrane device that modulates the excessive inflammatory response to sepsis. PloS One.

[66] Humes HD, Sobota JT, Ding F, Song JH, RAD Investigator Group (2010). A selective cytopheretic inhibitory device to treat the immunological dysregulation of acute and chronic renal failure. Blood Purif..

[67] Johnston KA, Pino CJ, Chan G, Ketteler SK, Goldstein SL, Humes HD (2023). Immunomodulatory therapy using a pediatric dialysis system ameliorates septic shock in miniature pigs. Pediatr. Res..

[68] Lee KH, Cavanaugh L, Leung H, Yan F, Ahmadi Z, Chong BH (2018). Quantification of NETs-associated markers by flow cytometry and serum assays in patients with thrombosis and sepsis. Int. J. Lab. Hematol..

[69] Langseth MS, Helseth R, Ritschel V, Hansen CH, Andersen GØ, Eritsland J (2020). Double-stranded DNA and NETs components in relation to clinical outcome after ST-elevation myocardial infarction. Sci. Rep..

