# Supplementary material for: Leukocyte filtration and leukocyte modulation therapy during extracorporeal cardiopulmonary resuscitation in a porcine model of prolonged cardiac arrest
Source: Sci Rep. 2024 Jun 7;14:13081. doi: 10.1038/s41598-024-63522-w (PMC11156900; doi:10.1038/s41598-024-63522-w)
Supplement: Supplementary file 1 — Supplementary Information. [file 41598_2024_63522_MOESM1_ESM.docx]

**
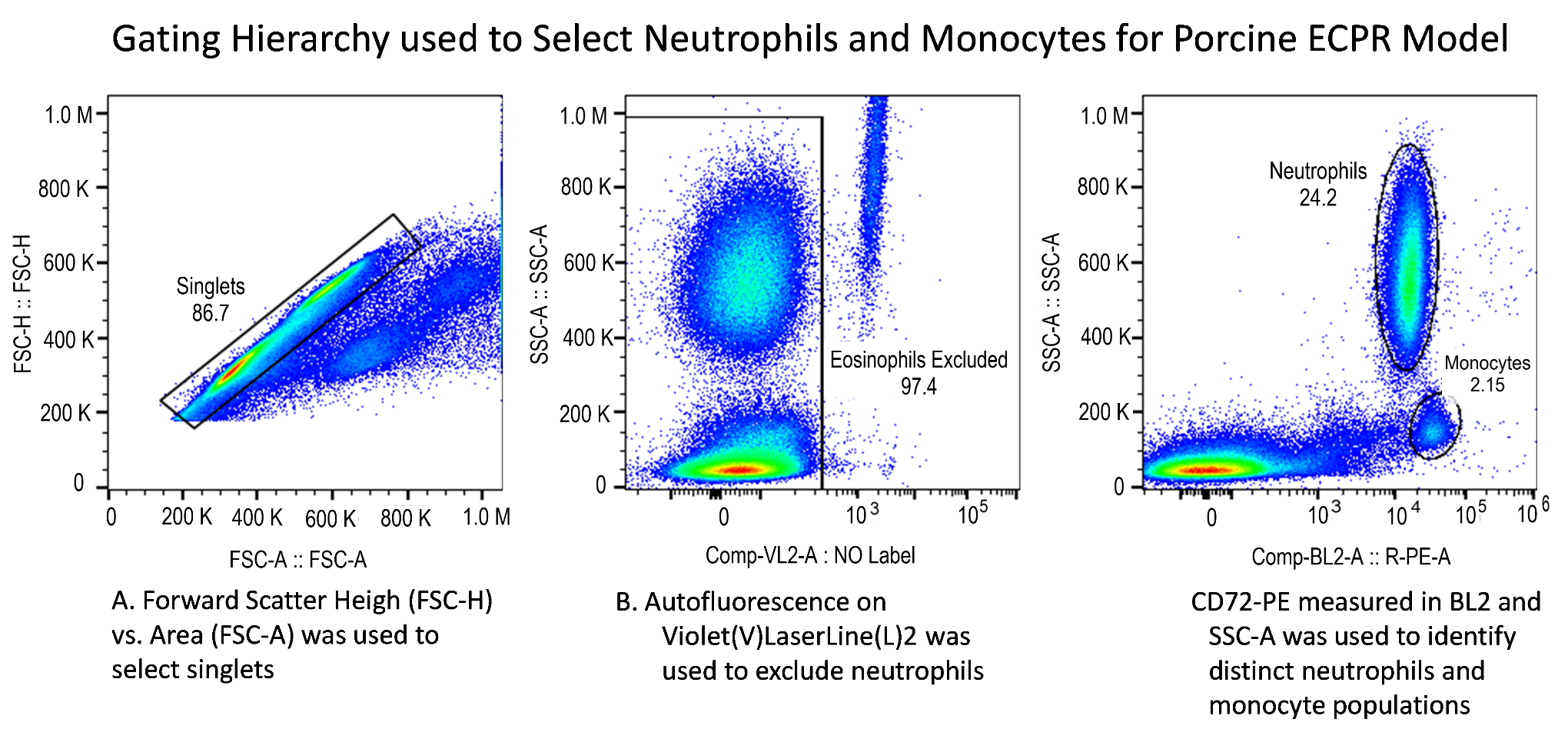
Supplementary Material**

**Supplementary Figure 1.** **Gating Hierarchy used to Select Neutrophils and Monocytes for Porcine ECPR Model.** A typical gating schematic is shown above. Forward Scatter Height vs. Width scatter plot is used to differentiate single cells from aggregates (A). Singlets are gated and eosinophils are separated from neutrophils based on autofluorescence (B). Eosinophils have an auto fluoresce an order of magnitude higher than neutrophils and if not differentiated eosinophil autofluorescence can falsely elevate the parameter of interest. It is important to separate these granulocytes because in this model neutrophil numbers decline disproportionately to eosinophils. Porcine monocytes and neutrophils are strongly positive for CD172a, and neutrophils vs monocyte granularity are employed to gate specifically for monocyte and neutrophil populations. Finally, neutrophils and monocyte mean fluorescence intensity (MFI) for CD11R3 was measured.

**
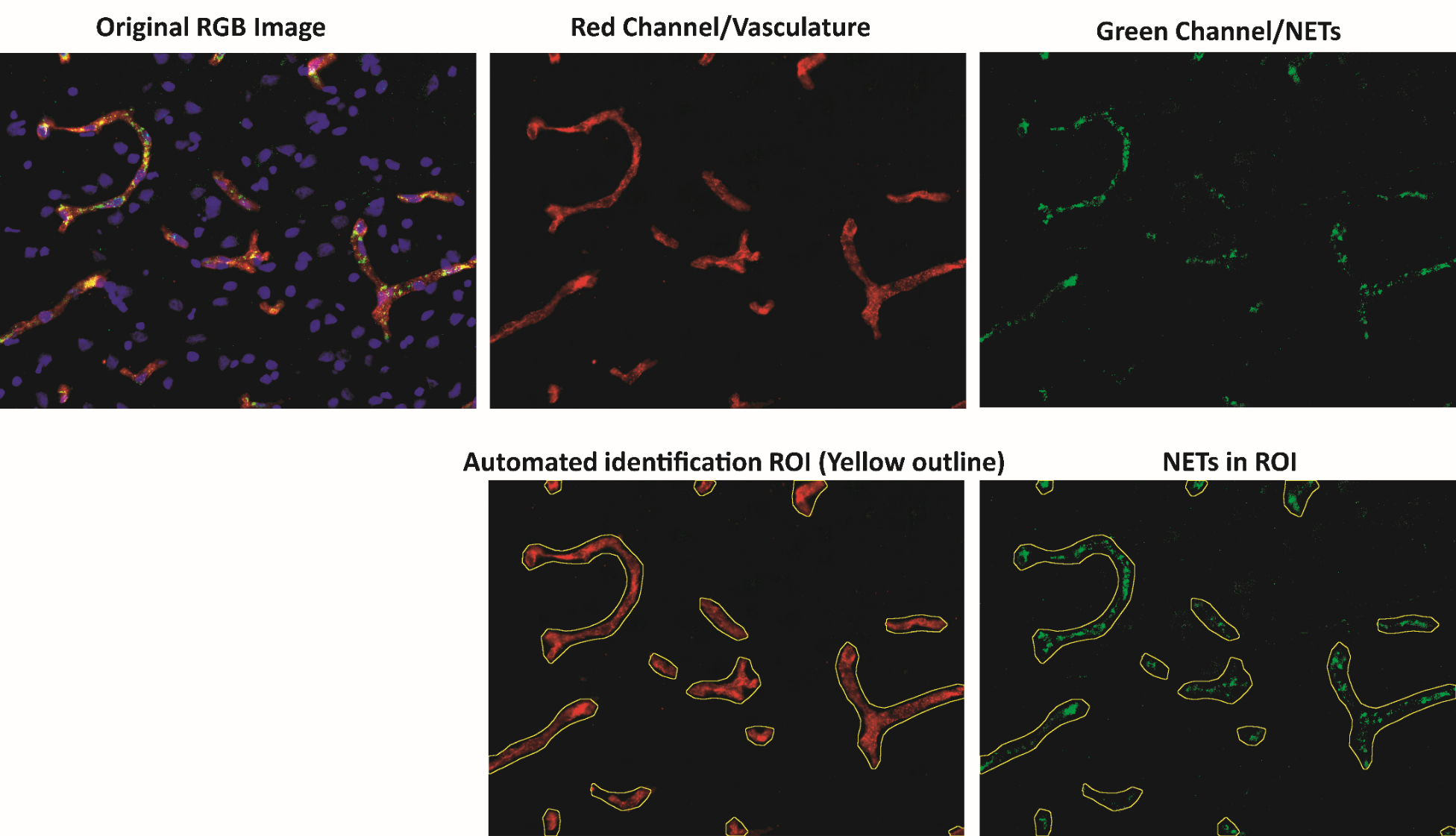
**

**Supplementary Figure 2. Automated Quantification of NET Formation with Fiji Image J.** The original RGB images (top left) were split into a red and green channel. The red channel (top middle) shows the vasculature, and the green channel (top right) shows the NETs. The software identifies vasculature well in the red channel as the region of interest (ROI), which is outlined in yellow (bottom middle). The NET signal is colocalized with vasculature (bottom right). The NET formation is quantified as the percentage of vasculature.

**A. Myocardial Histopathology**

|  | **Naïve, n=4** | **Control, n=8** | | **LF, n=12** | | **L-MOD, n=12** | |
| --- | --- | --- | --- | --- | --- | --- | --- |
| Myofiber degeneration, median (IQR) | 1 (0.85-1.25) | 1.85 (1.35-2.05) | | 1.75 (1.2-1.9) | | 1.9 (1.35-2.15) | |
| Myocardial hemorrhage, median (IQR) | 0 (0-0.05) | 0 (0-0) | | 0 (0-0) | | 0 (0-0) | |
|  |  | |  | |  | |  |

**B. Neuronal Histopathology**

|  | **Naïve, n=8** | **Control, n=8** | **LF, n=12** | **L-MOD, n=12** |
| --- | --- | --- | --- | --- |
| Caudate degeneration, median (IQR) | 0.9 (0.25-1.05) | 1.7 (0.8-2.15) | 1.85 (1-2.3) | 1.5(0.8-1.95) |
| Hippocampus degeneration, median (IQR) | 0.45 (0.3-1) | 0.8 (0.4-1.05) | 0.75 (0.4-0.8) | 0.5(0.3-1) |
| Cingulate degeneration, median (IQR) | 0.45 (0.3-1.55) | 1 (0.75-1.65) | 1.85 (1.1-2.05) | 1.55 (0.85-2.05) |
| Cerebellum degeneration, median (IQR) | 0.7 (0.35-0.95) | 0.2 (0.05-0.8) | 0.55 (0.15-0.9) | 0.15 (0.05-0.75) |

**C. NET Formation in Heart**

|  | **Naïve, n=4** | **Control, n=8** | **LF, n=12** | **L-MOD, n=12** |
| --- | --- | --- | --- | --- |
| NETs (% vasculature), median (IQR) | 3.23 (1.70-4.52) | 6.51 (3.46-8.92) | 5.53 (2.92-8.76) | 4.28 (1.93-6.71) |

**D. NET Formation in Brain**

|  | **Naïve, n=8** | **Control, n=8** | **LF, n=12** | **L-MOD, n=12** |
| --- | --- | --- | --- | --- |
| NETs (% vasculature), median (IQR) | 2.11 (1.39-4.23) | 9.16 (3.92-26.45) | 4.96 (2.02-6.15) | 10.58 (2.11-27.58) |

**Supplementary Table 1. Histopathology and NET Formation in Brain and Heart.** Myocardial (A) and neuronal (B) histopathology quantifications are presented in the table below as median (IQR). NET formation quantified as a percentage of the vasculature in the heart (C) and brain (D) are presented in the table below as median (IQR).
